# Computational Study of Correlated Domain Motions in the AcrB Efflux Transporter

**DOI:** 10.1155/2015/487298

**Published:** 2015-01-05

**Authors:** Robert Schulz, Attilio V. Vargiu, Paolo Ruggerone, Ulrich Kleinekathöfer

**Affiliations:** ^1^School of Engineering and Science, Jacobs University Bremen, Campus Ring 1, 28759 Bremen, Germany; ^2^Dipartimento di Fisica, Università di Cagliari, S.P. Monserrato-Sestu km 0.7, 09042 Monserrato, Italy

## Abstract

As active part of the major efflux system in *E. coli* bacteria, AcrB is responsible for the uptake and pumping of toxic substrates from the periplasm toward the extracellular space. In combination with the channel protein TolC and membrane fusion protein AcrA, this efflux pump is able to help the bacterium to survive different kinds of noxious compounds. With the present study we intend to enhance the understanding of the interactions between the domains and monomers, for example, the transduction of mechanical energy from the transmembrane domain into the porter domain, correlated motions of different subdomains within monomers, and cooperative effects between monomers. To this end, targeted molecular dynamics simulations have been employed either steering the whole protein complex or specific parts thereof. By forcing only parts of the complex towards specific conformational states, the risk for transient artificial conformations during the simulations is reduced. Distinct cooperative effects between the monomers in AcrB have been observed. Possible allosteric couplings have been identified providing microscopic insights that might be exploited to design more efficient inhibitors of efflux systems.

## 1. Introduction

Antibiotic resistance is a challenging problem to the health care sector [1, 2]. Especially multidrug-resistant (MDR) bacteria increase the frequency of therapeutic failure [3]. Only few new antibiotics are brought to market these days and the understanding of where resistance originates might give a new boost to the development of new drugs [2]. As an important step before antibiotics can be effective, the membrane of the bacteria has to be passed. This may already pose a formidable problem as some bacteria might only express narrow pores or mutations might lead to decreased expression of porins, porin loss, or narrow channels [4]. At the same time bacteria contain efflux systems that shuttle certain substrates out of the cell. In the case that certain antibiotics are recognized as substrates for those efflux pumps, these antibiotics become basically ineffective for that specific bacterium [5–7].

A detailed molecular understanding of antibiotics transport through the cell envelope [8], that is, influx and efflux, would offer new opportunities for drug discovery [6, 7]. In* Escherichia coli* the AcrAB-TolC multidrug efflux pump containing a transporter of the resistance-nodulation-cell-division (RND) family plays a major role in the intrinsic and acquired resistance to multiple classes of structurally distinct antimicrobials [9–12]. The AcrAB-TolC system has been studied intensively in recent years and structural data for all involved proteins are available [13]. The tripartite complex consists of an active RND transporter embedded in the inner membrane, AcrB [9, 14, 15], an outer membrane exit channel in the outer membrane, TolC [16, 17], and a periplasmic membrane-fusion protein, AcrA [18]. Substrates are supposed to be taken up from the periplasm and the outer leaflet of the inner membrane into the transporter AcrB. From there they will be pumped through the channel TolC out of the cell. The membrane-fusion protein is stabilizing the complex of AcrB and TolC. Details of the assembly are still unclear though an increasing number of aspects have been unravelled recently [19–21].

**Figure 1:**
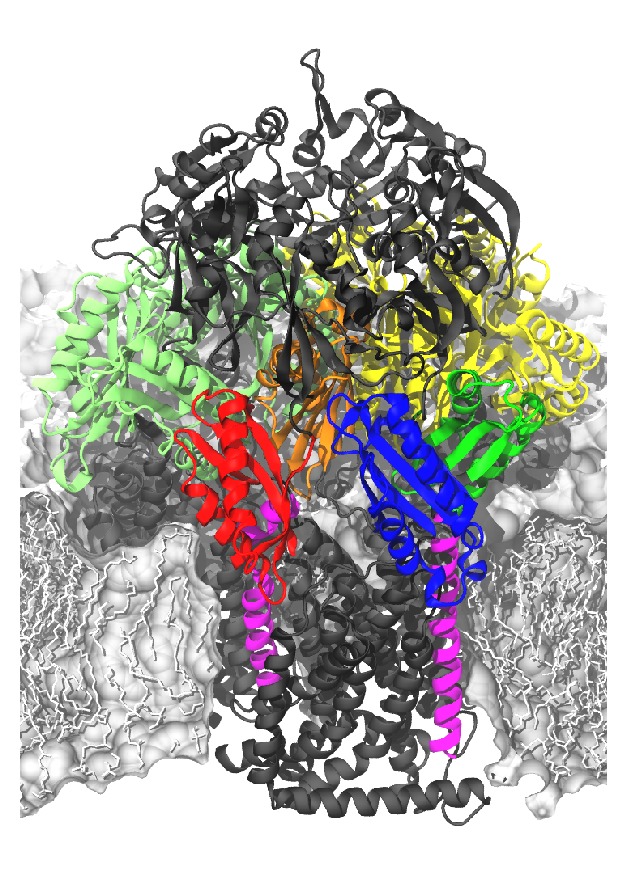
Simulated system with AcrB (in black together with highlighted regions in additional colors) embedded in a lipid bilayer (in grey). The light green and yellow colored domains in the background denote the porter domains of L and O monomers, respectively. The subdomains of the porter domain in the foreground (monomer T) are colored individually: PC1 blue, PC2 red, PN1 orange, and PN2 dark green. In addition, transmembrane helices 2 and 8 of T monomer (left and right helices, resp.) are colored magenta.

Structural data allowed for molecular level simulations of the individual parts of the efflux system [22, 23]. The outer membrane protein TolC has been studied [24, 25] as well as AcrA [26] and the* Pseudomonas aeruginosa* AcrA-homolog MexA [27]. In the present study, however, we focus on the active transporter AcrB (see Figure 1), which has been simulated already in previous studies by the present authors [28–30] and others [31, 32]. Based on available crystal structures and biochemical data, the transport of substrates by AcrB has been proposed to take place via a functional rotation, in which each monomer neatly assumes in a succession of steps each of three particular conformations [9, 14, 33], labeled as L (loose), T (tight), and O (open) according to Seeger et al. [9].

For a computational study of the functional rotation conventional molecular dynamics (MD) simulations are not feasible owing to the time scale of the process and the size of the systems. To enforce conformational motions in proteins during simulations, the targeted molecular dynamics (TMD) simulation scheme has been developed [34] and successfully applied to study conformational changes in large systems such as F_1_-ATPase [35], MurD [36], G-proteins [37], and the transporter BtuCD [38]. Additionally, the approach can be employed to provide reliable transition paths similar to other methods used to sample conformations of proteins [39] though problems with large-scale motions preceding small-scale motions are well known [40]. In previous investigations [28] we used the TMD approach to mimic the functional rotation of the transport protein AcrB. These conformational changes induced a detachment of the substrate from the distal binding pocket (DP) [14, 15, 41] and a movement towards the exit gate of the protein. Furthermore, these TMD simulations provided strong evidence for the earlier proposed peristaltic transport involving a zipper-like closure of the binding pocket. This movement is in turn responsible for the displacement of the drug in the direction of the gate, which is a crucial passageway during the translocation of the drugs from the DP towards the TolC channel. A concerted opening of the channel between the DP and the gate further favors the displacement of the drug.

Similar computational protocols were used by some of the authors to gain insights into the role of water molecules in this extrusion process [29], the influence of point mutations on the affinity of a compound to the transporter [30], the interaction of several substrates, nonsubstrates, and inhibitors with AcrB [42], and the recognition of imipenem and meropenem by MexB, the homologous protein of AcrB in* P. aeruginosa* [43]. These studies have demonstrated the maturity of such computational strategies in teasing out atomistic insights into the functioning of the complex RND transporters.

The present computational investigation is not focused on the characterization of the movement of the drug in the AcrB transporter but more specifically on the identification of possible correlated motions of different parts of the protein. Similar studies have been performed by using unbiased MD simulations to deepen aspects related to interdomain movements in polyketide synthases [44] or elastic network models to gain insights into the AcrAB-TolC complex [45]. For example, in the latter work, based on a simplified model of the protein, Wang and coworkers observed conformational couplings across monomers in the AcrB trimer. Our study relies on the analysis of trajectories extracted from all-atom MD simulations. Firstly, we considered a 200 ns long unbiased MD simulation of AcrB in order to look for correlated motions. In a second step we analyzed several TMD simulations in which either all C_*α*_ atoms of the whole protein scaffold or only specific parts of the protein such as the transmembrane domain or neighbouring monomers were forced. In all these TMD simulations the starting state of the trimer is the so-called L-T-O state and the target state T-O-L. A detailed correlation analysis of the atomic movements yields interesting results concerning cooperative effects in the transporter and concerning intermonomeric couplings. The insights gained through these analyses might pave the way to the identification of possible allosteric sites and links that can be of interest to the design of efficient inhibitors. At the same time the present investigation shows the advantages but also limitations of TMD simulations in which large conformational motions, usually beyond the time scale accessible by MD simulations, are enforced on a time scale of several tenths of nanoseconds. 

## 2. Results

Table 1 lists the different steering selections together with the respective MD simulation times. For the sake of clarity, some particular regions of interest are highlighted in Figure 1.

As described in previous publications on the crystal structures [9, 14, 46], the transporter AcrB can be clearly divided into (a) three monomers or (b) three domains along the vertical axis, that is, transmembrane domain, porter domain, and TolC-docking domain. Hence, the selections of steered protein segments were derived accordingly. As already mentioned, in all TMD simulations the starting state of the trimer is the so-called L-T-O state and the target state T-O-L.

### 2.1. Setups for the Simulations

In a first step, the equilibrium dynamics of the 200 ns long unbiased AcrB simulation (*freeDyn*) was analyzed to find prominent conformational changes occurring without any forces applied. In a second step, the steering of the transmembrane domain,* tmDom*, was investigated to find possible clues on the transduction of mechanical energy. Based on structural evidence it has been proposed that the proton gradient across the membrane induces a conformation change in transmembrane helix 8 (abbreviated as* tmH8* hereafter) [15]. This helix is thought to be responsible for the major transmission of mechanical energy from the transmembrane to the porter domain leading to a closure of the entrance in the porter domain during the transition T → O [9, 14, 46]. Therefore we enforced conformational changes in the transmembrane domain and the resulting configurational rearrangements and effects on the porter domain were examined.

The focus in the present study is on monomer T, that is, the monomer loaded with doxorubicin, as in our previous studies [28, 29]. To better understand the motions of this monomer, in a third setup only the neighboring monomers of the T monomer were steered exclusively (*freeMon*). To this end we used the same initial conformation as in the previous investigations. This simulation is done in order to clarify the influence of the neighboring monomers on the unsteered T monomer in the T → O transition. Finally, both selections,* tmDom* and* freeMon*, were combined resulting in simulations where both, the neighboring L and O monomers as well the transmembrane domain of monomer T, were steered and only the porter and TolC-docking domains of the occupied monomer are free to move (*freePP*).

**Figure 2:**
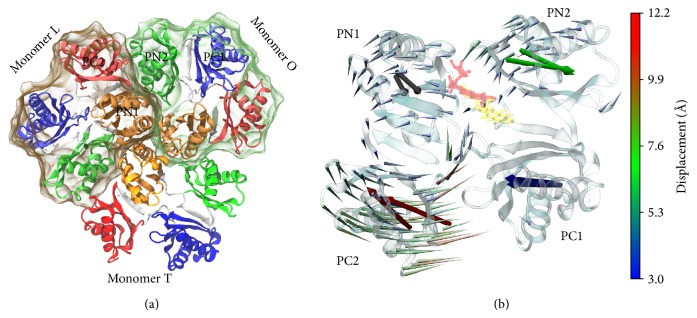
(a) The subdomains of the entire porter domain colored and labelled individually. The monomers are separated by shaded surfaces. (b) Expected movements of the subdomains belonging to monomer T. The arrows of the so-called porcupine plot [24] indicate the linear deviations between the initial and final states of the T → O transition. The longer rod-like arrows describe the movement of the principal axes of each subdomain in the direction of the transition depicted by the porcupine representation. The initial (yellow) and final (pink) positions of the drug molecule are taken from a* fullTMD* simulation.

The subdomains of the AcrB porter domain together with the proposed conformational changes in monomer T during the T → O transition are shown in Figure 2. The latter changes have been derived from a* fullTMD* simulation in which all C_*α*_ atoms of the whole protein have been steered. For simulations in which only a smaller selection of atoms is forced, these results can serve as benchmark. Moreover, we would like to note in passing that though often not explicitly mentioned, the transition T → O should be understood as L-T-O → T-O-L; that is, also the two other monomers change their conformations according to the proposed cycle.

### 2.2. The Protein Dynamics in Unbiased and Fully Biased Simulations

Before going into details about the individual partial-TMD simulations, the intrinsic flexibility and motion of the protein are derived from the 200 ns long unbiased MD simulation* freeDyn*. This run started from the same configuration as all the other partial-TMD and* fullTMD* simulations (see* Methods* for more details).

**Figure 3:**
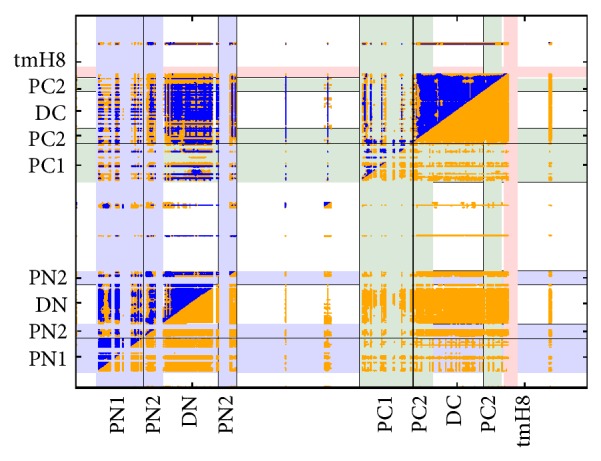
Comparison of two methods to obtain spatial correlations for the T monomer in the unbiased simulation* freeDyn*. In both symmetric triangular parts of the graph each yellow point corresponds to a Pearson correlation coefficient *ρ*_*P*_ greater than 0.5. In the upper left triangular part each blue point has a generalized correlation coefficient *ρ*_gen_ greater than 0.5. Correlation values are only displayed if the RMSF of the related residues is larger than 1.5 Å. The lightly colored regions describe the periplasmic subdomains PN1 + PN2 (blue—residues 42 to 177 and 287 to 325) and PC1 + PC2 (green—residues 571 to 721 and 822 to 859), respectively, as well as the transmembrane helix 8 (red).

To examine correlated motions between different segments of the protein, we calculated the correlation matrix of the coordinates belonging to all C_*α*_ atoms along the trajectory. In Figure 3, the intramonomeric correlations for the T monomer are displayed in matrix style. In all correlations shown throughout this paper, a threshold of 1.5 Å is employed to emphasize those residues that are actually moving significantly during the simulation. In Figure 3, we compared the standard Pearson correlation coefficient *ρ*_*P*_ with the linear generalized correlation coefficient *ρ*_gen_ introduced by Lange and Grubmüller [47] as well as Kraskov et al. [48]. For simplicity all correlation coefficients whose absolute values are lower than 0.5 were neglected in Figure 3 and hereafter.

In Figure 3 strong correlated motions are visible within the TolC-docking domain parts DN (the region of the graph comprised between the two sections of PN) and PN and their directly linked protein parts in the porter domain, that is, PN2 and PC2. These correlations are noticeable close to the diagonal in the graph. Even more interesting are the strongly correlated motions between different subdomains. Strong correlations are observed between the PN and DN parts of the TolC-docking domain. These correlated motions also extend to the connected parts in the porter domain but to a somewhat lesser degree: DN is correlated with PC2 and DC with PN2. Interestingly, both subdomains in the TolC-docking domain are well connected, thereby leading to a rather high correlation. With these results one has to keep in mind that the Pearson coefficient as a measure for correlation has a serious shortcoming though it is applied in many studies. As detailed in [47], the Pearson correlation only takes into account correlations between collinear motions whereas the linear generalized correlation coefficient also includes other types of correlated motions partially leading to quite different results. Therefore, the results of this comparison can roughly be described as *ρ*_*P*_ being a subset of *ρ*_gen_ though the numerical scales of the two correlation measures are not directly comparable. Because the generalized correlation yields a more general perspective onto the correlated protein motion, it is the one which will be used in the following analyses.

**Figure 4:**
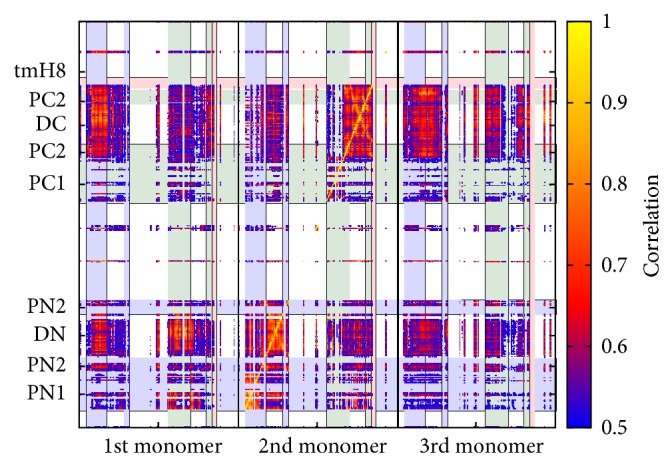
Comparison of intermonomeric generalized correlation coefficients *ρ*_gen_ between the T monomer (2nd monomer) and the other three monomers for the unbiased simulation. The applied limits as well as the colored regions indicating specific protein regions are set as in Figure 3.

In Figure 3 the information on the intramonomeric interactions was coarse grained in the sense that only correlation coefficients larger than 0.5 are depicted.  Figure 4 shows the intermonomeric correlated motions between monomers L, T, and O including the intramonomeric correlation of monomer T using a color representation. A stronger correlation is observed within the T monomer if compared to the neighbours and an equally remarkable correlation marks the TolC-docking domain. This correlation is due to concerted motions between the DN and DC subdomains. In conclusion of this correlation study, significant correlated motions within but also between monomers of AcrB are clearly visible and can be identified in well-defined regions of the system.

### 2.3. Intramonomeric Interactions during Partial-TMD Simulations

On the basis of the crystallographic data for AcrB [9, 14, 46] it was postulated that the functional rotation is associated with a mechanical transduction of the energy stored in the transmembrane domain because of the proton flux toward the porter domain. In particular, Sennhauser et al. [46] presented a scheme, which highlighted the essential conformational changes including those of helix* tmH8* related to the efflux process. The helix* tmH8* is one of two extended helices (the other is helix 2,* tmH2*) protruding from the transmembrane domain farther toward the porter domain. Analyzing the conformational differences between the states of the functional rotation (derived from asymmetric crystal structures),* tmH8* moves more prominently while* tmH2* translates only slightly [15]. Hence, the behaviour of* tmH8* was addressed in more detail. At the same time,* tmH8* together with* tmH9* is speculated to form a possible entry pathway in the L monomer for substrates that are partitioned in the outer leaflet of the inner membrane [15].

**Figure 5:**
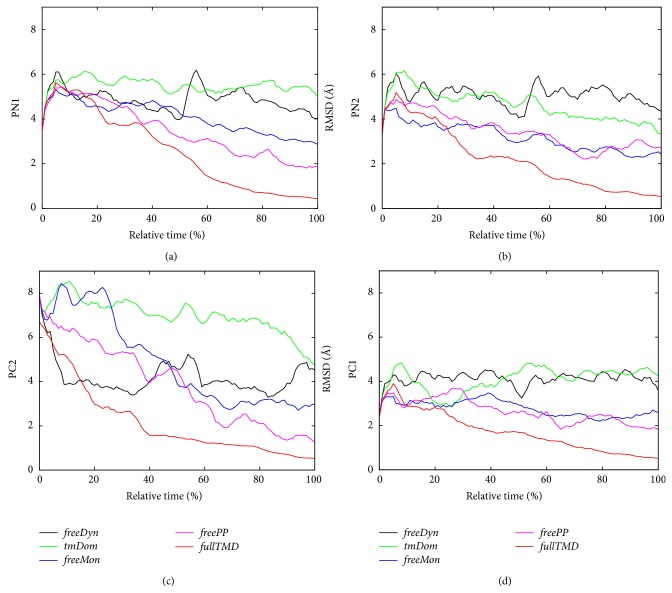
RMSD of the C_*α*_ atoms belonging to the T monomer of the initial state shown for different TMD selections. The deviation is determined with respect to the target state, that is, the next step in the functional rotation, for simulations progressing from the initial to the target state. A running average of 20 simulations steps was applied here as well as in the following graphs.

To quantify structural changes in the protein we calculated the root mean square deviations (RMSDs) of the porter subdomains with respect to the TMD target state T-O-L (L-T-O is the starting state) and the obtained curves are collected in Figure 5. The decreasing trend of the curves is due to the fact that the reference state is the final state of the TMD simulations. For* freeDyn* RMSDs (black curves in the panels) of PC1, PN1, and PN2 mainly fluctuated around the starting value, as expected. Only for PC2 we observed a clear decrease, which might indicate that the PC2 domain was not in its equilibrated conformation at the beginning of the simulations (see also Section 3). For the fully steered simulation,* fullTMD*, the RMSDs (red curves) approached the zero value at the end of the simulation time. This value is usually not reached in TMD simulations due to the finite spring constant employed in the TMD approach. The flexibility of the spring allows for differences between the target and the actually reached conformations. If only the transmembrane domain is steered in the* tmDom* setup (green curves), PC1, PN1, and PN2 displayed RMSDs similar to those extracted from the unbiased trajectories. PC2 departed initially from the behaviour observed in* freeDyn* with an increasing RMSD. However, at the end of the simulation the curve for* tmDom* obtains a value close to the one for the unbiased trajectory.

In* freeMon* (blue curves) only the monomers L and O and not T were forced to undergo the conformational change of the functional rotation. The blue curves in Figure 5 indicated that structural changes in the T monomer were significant but not as large as those observed in the fully biased simulations. Interestingly, by adding the transmembrane domain of the T monomer to the steered portions of the system (*freePP* simulation, magenta curves) no remarkable changes in the RMSD curves were observed with respect to* freeMon*. The domains PN1 and PC2 show slightly smaller RMSD values than those for the* freeMon* simulation while the values for the domains PN2 and PC1 are very similar. As a major finding we state that it is obviously not enough to only steer the transmembrane domain in short TMD simulations to observe differences, as demonstrated by the fact that results of* freePP* and* freeMon* simulations behave similarly. At the same time, steering the residues of the neighbouring monomers leads to significant conformational changes toward the target state different from the unbiased case.

**Figure 6:**
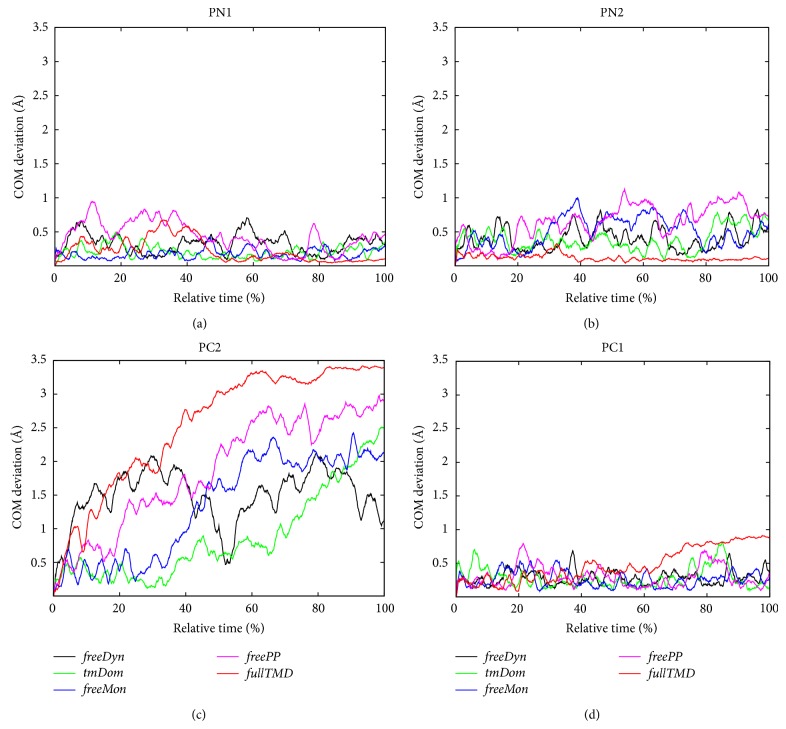
Translational movement of the centers of mass (CoMs) of the four subdomains during the different types of simulations.

To quantitatively describe the actual displacement of the individual sections of AcrB in the simulations we evaluated the center-of-mass (CoM) displacements of the subdomains, which are reported in Figure 6. For three out of the four subdomains no distinct direction of transition can be observed. Only PC2 shows a significant deviation of more than 1 Å for all simulations. To clarify that this is not simply due to the fact that PC2 could be the subdomain with the smallest mass, the number of atoms and the corresponding masses were determined. Subdomain PC1 contains 837 atoms, PC2 608 atoms, PN1 671 atoms, and PN2 579 atoms with the total masses of these subdomains being roughly proportional to the number of atoms. Hence, this measure does not seem to be very informative to quantify the subdomain motions. As alternative, we analyzed the orientations of the subdomains by evaluating the rotational movement of the major principal axis (PA) of the regions. The PAs are defined as the major PAs of the moment of inertia tensor of the respective subdomains and calculated using VMD [49]. The angle Φ is determined by projecting the movement of the major PA onto the membrane plane. Moreover, the angle with respect to the membrane normal is called Θ. Both angles are defined with respect to the initial conformation (for a graphical representation see Figure S5 in [29]). Using these angles in addition to the COM motion of the subdomains one can describe their overall movement more accurately.

**Figure 7:**
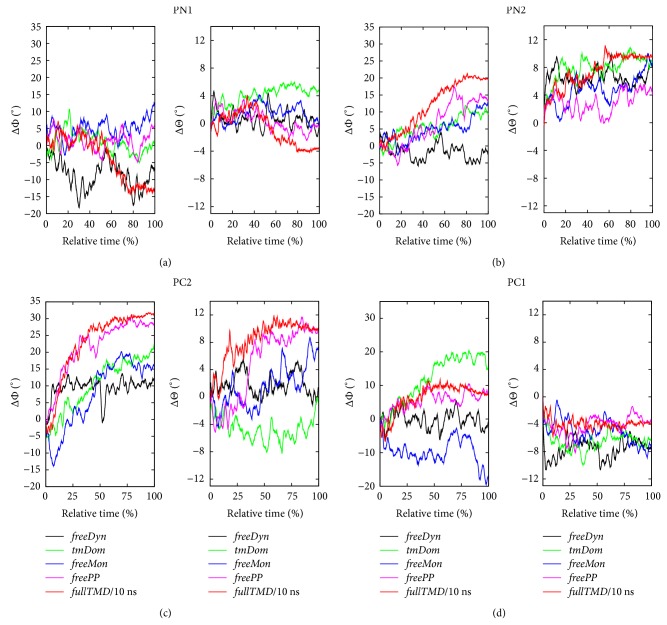
Rotation of the four subdomains for the different simulations in the same order as Figure 2. The described spherical angles are of the same type as described in [29].

While, in the* fullTMD* simulation, the protein was driven along a rather distinct path by the TMD forces, the other simulations are characterized by more enhanced flexibility as shown in Figure 7. Despite the less pronounced transitions of some of the subdomains compared to the* fullTMD* reference trajectory, major conformational changes especially in subdomains PC2 and PN2 were observed. While, for PC2, a distinct CoM displacement and rotation of the Θ angle was measured, PN2 in addition undergoes a rotational movement in both angles. Note that the latter subdomain did not show any significant CoM displacement during the* fullTMD* simulation. The Φ angle of PC2 did change by 20° in simulation* fullTMD* compared to a 10° change in the* tmDom* trajectory independently of the simulation time. Furthermore, the results concerning the Θ angle of PC2 did not vary substantially with the trajectory length. Other than minor changes in the Φ angle, significant conformational changes did not occur in the PN1 domain during the* tmDom* simulation. Apart from PN2, only the Φ angle of PC2 shows a distinct direction of change in all steered trajectories, which is probably due to the extended* tmH8* helix and to the spatial proximity of the PC2 and transmembrane domains. The observed transitions of PN2 have smaller amplitudes than those of PC2 but do not seem to be dependent on the specific TMD selection. The comparison of the steering schemes* tmDom* and* fullTMD* indicated a correlation between the extrusion of the substrate from DP and the transition of PN2 (data not shown).

### 2.4. Intermonomeric Interactions

To estimate the influence of the neighboring monomers on the conformational state of the examined monomer, we compared in more detail the* freeMon* and the* freePP* simulations but also the* tmDom* variant. Please remember that in the* freePP* simulation the transmembrane domain is steered in addition to the neighboring monomers, that is, the* freeMon* simulation. As in the previous section, we focused on the orientational changes reported in Figure 7. A striking aspect of these orientational parameters for* freeMon* and the* freePP* is their proximity to the reference data from the* fullTMD* simulation. Especially for the PN1 and PN2 subdomains the* freeMon* and the* freePP* simulations behave very similarly. For the PC1 and PC2 subdomains, however, the additional steering of the transmembrane domain leads to a better agreement with the* fullTMD* simulation thanonly steering the neighboring monomer.

Referring back to Figure 5, the conformational transitions of both major setups,* tmDom* and* freePP*, are compared more quantitatively using the RMSD values, evaluated by using two setups as references, namely, the 200 ns long unbiased simulation* freeDyn* and a sample trajectory of* fullTMD*. While the general idea of “the more parts you steer, the closer the results are to* fullTMD*” still seemed to be true for most of the cases, some trajectories in this figure deviated from this expectation. For instance, the RMSD of PN2 did not appear to depend on the TMD selection. In fact, the 50 ns* freePP* simulation showed a slight increase of RMSD at the end of the trajectory, that is, at *t*_*rel*⁡_ ~ 80%. Moreover, the RMSD of subdomain PC2 was lowered more largely for the* freeDyn* setup than for* tmDom*. Only the RMSD of the 50 ns* freePP* simulation displayed the same trend as the* fullTMD* trajectory.

### 2.5. Methods

Since the simulation protocol is the same as that in our previous studies [28–30] we only list some major features here. Both of the unbiased MD and the TMD simulations were performed using the parallel MD code NAMD 2.7b1 [50]. For all amino acids, their standard protonation states were considered, that is, the states as for pH 7. After an equilibration procedure [28–30] the MD simulations were performed with a 1 fs time step in an NpT ensemble at 310 K and 1.013 bar. The functional rotation was enforced using TMD [34] (built-in module of NAMD) which allows inducing conformation changes between two known states. In the present investigation different parts of the protein were steered using this approach. The force constant per atom was chose to be* k* = 3 kcal/(mol Å^2^). The setup, the analyses, and the atomic-level figures were performed using VMD [49].

To investigate the intra- and intermonomeric interactions, correlation matrices have been calculated using the program* g_covar* from the Gromacs package [51]. This tool computes the Pearson correlation of a set of atoms, in this case of all C_*α*_ atoms of the protein. Reference [47] describes this approach as inapplicable to study three-dimensional protein systems since the Pearson correlation does only consider colinearly correlated motions of two atoms. Hence, more elaborated correlations cannot be estimated using this method. Therefore, Lange and Grubmüller [47] developed a new method which they called “generalized correlation” and which is supposed to be able to cover these correlations as well and has been applied in the present study.

## 3. Discussion and Conclusion

In the present work, we focused on examining the usage of TMD simulations by considering various selections of steered protein segments. While the TMD approach [34] has been applied to all residues of the protein in previous studies [28, 29], it is utilized on specific domains or monomers of AcrB in the present contribution. Although the time scale of the MD simulations is limited to 50 ns and 200 ns here, these theoretical investigations help to pinpoint possible dependencies and couplings for subdomain transitions.

Concerning the fluctuations of the protein we looked at them as a function of amino acid sequence initially. Subsequently, the RMSF values were mapped back onto the structure showing that the linked PC2 and PN1 are obviously more stable at their interface than at the more distant segments (data not shown). Interestingly, the least fluctuating parts of the porter domain are PC1 and the inward facing beta sheets of PN1 and PN2. While PC1 was previously stated as rather static, PN2 is supposed to be a particularly flexible subdomain which opens and closes the binding pocket. Furthermore, subdomain PN1 is strongly linked to PC2 and regulates the exit gate. To overcome the limitations of the RMSF measure, we analyzed the motions and orientations of specific subdomains.

**Figure 8:**
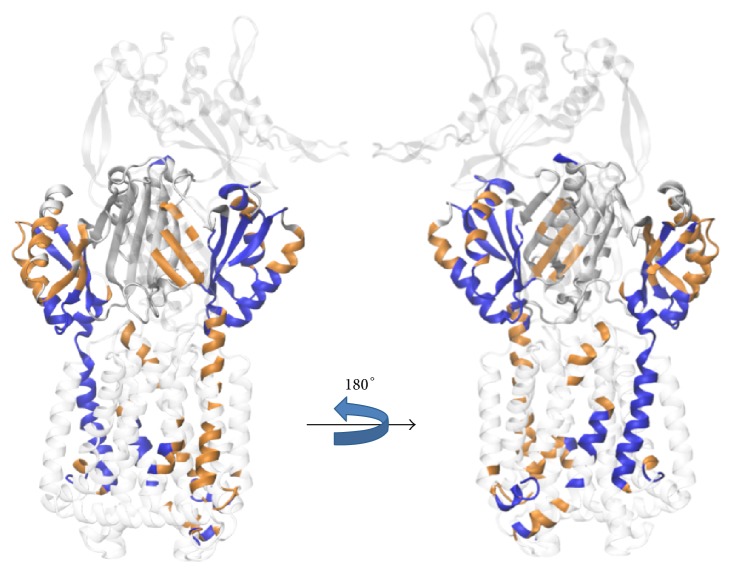
Correlation matrices based on the simulation* freePP* mapped back onto the protein structure. The porter domain is drawn in grey and the other domains in transparent. The correlation highlights are drawn as an overlay on top of these. The color code is the same as that in Figure 3 and the structure is shown from two opposite sides.

An interesting aspect is that the coefficients concerning the correlated motions can be mapped back onto the structure by highlighting all parts of the structure contributing to correlation coefficients larger than 0.5 as shown in Figure 8. A strong correlation is observed within the T monomer if compared to the neighbours and an equally remarkable correlation characterises the TolC-docking domain of all three monomers. This domain seems to keep the entire trimer in shape. Part of this effect is facilitated by the extended arm reaching from the DN subdomain toward the neighboring monomer (see above). In comparison to the DN subdomain, the DC subdomain does not show so highly correlated motions with the other subdomains. These aspects of the TolC-docking domain have to be seen in the context that the porter and the transmembrane domains are linked by peptide bonds only at four different points per monomer. The interface between these two latter domains mainly contains unstructured loops, rendering the connection between porter and transmembrane domain quite flexible.

As expected, the long unbiased simulation* freeDyn* showed basically only fluctuations around the initial structure. For the PC2 subdomain, however, we observed a clear movement away from that initial structure indicating that the starting conformation close to the crystal structure may not be the equilibrium structure of the complete complex. As pointed out by Fischer and Kandt [32] the structure of AcrB did not reach a complete equilibrium after 100 ns of simulations. For sure, a complete picture of the possible couplings requires remarkably longer simulations. However, we believe that indications on possible linkages and time scales of correlations can be extracted from the present simulations. As it can be seen in Figure 5, if only the transmembrane domain is steered in the* tmDom* setup, PC1, PN1, and PN2 displayed RMSDs similar to those extracted from the unbiased trajectories. PC2 departed initially from the behaviour observed in* freeDyn* with an increasing RMSD. However, at the end of the simulation the* tmDom* curve approached the unbiased one. This indicates that the transduction of mechanical energy from the transmembrane domain towards the porter domain seems to need much more time than the 50 ns of the TMD simulation. Note that shorter test simulations of the* tmDom* variant did not necessarily lead to results further away from those of the* fullTMD* type. This hints at a possible lack of other probably intermonomeric contributions, facilitating the transition between the states of the functional rotation cycle.

In simulation* freeMon* only two of the three monomers were forced to undergo the conformational change of the functional rotation: monomers L and O were steered but not T. The blue curves in Figure 5 indicated that structural changes in the T monomer were not as large as those observed in the fully biased simulations but had the same trend. This points toward the fact that there are large cooperative effects in the domain motions between the different monomers.

Interestingly, by adding the transmembrane domain of the T monomer to the steered portions of the system (*freePP* simulation) no remarkable changes in the RMSD curves were observed with respect to* freeMon*. Only for PC2,* freePP* indicates lower RMSD values towards the end of the trajectory. This is again an argument for the importance of intermonomeric interactions during the functional cycle. Additionally, the lack of remarkable difference seems to define a lower boundary of 50 ns in the time scale over which the transduction of mechanical energy from the transmembrane to the porter domain occurs.

While the movement of PC2 induced by helix* tmH8* was already suggested in the literature [15], the conformational changes of PN2 were surprisingly unrelated to the actual TMD selection. Moreover, PN1 seems to require a defined interaction with the neighboring monomer since it does not show any major movement unless the neighboring domains are steered. In general, the majority of subdomain movements were mainly present in changes of their orientation rather than translation. Moreover, the initial state of PC2 which was derived from the crystal structure is obviously an extreme case since in the unbiased MD simulation* freeDyn*, used as control, the RMSD of PC2 from the O state was reduced by almost 50%. Only by combining the analysis of RMSD and CoM motion and orientation, the obtained data seem helpful in visualizing the correlations during certain transitions. Nevertheless, this selective version of the TMD method offers new possibilities to study protein transitions, especially if the general direction of energy transduction is known already which helps to define advantageous selections of steered residues.

In future studies, we believe that the partial TMD approach can be used in conjunction with unbiased simulations of different crystal structures, for example, ATP binding cassettes, to gather more information about domain motions and interactions during hypothesized transitions. At the same time the present study clearly showed the limitations of TMD simulations. Using a TMD simulation to steer in the limited simulation time the transmembrane domain in which an initial force is generated by the proton gradient is not enough to lead to significant conformational motions in the porter domain though this scenario can clearly be deduced from experimental findings. The same is true for forcing the helices, especially helix* tmH8*, which are supposed to transfer the forces between these domains. The finite-time TMD simulations apparently do not produce the conformational changes leading to the target structure. At the same time this might indicate that in addition to the helices other collective motions, for example, of neighboring domains, are needed to reach the target conformation though we cannot rule out that the time scales of our simulation were simply too short. Additionally, it should be pointed out that we simulated a single part of the efflux system. The influence of other components in the transmission of movements, such as AcrA or MexA, cannot be ruled out.

In conclusion, the present MD simulations and their analysis have shown that strong correlated motions within but also between monomers of the transporter AcrB do exist. Based on conventional and steered simulations we identified which subdomains within a monomer do strongly move in a correlated fashion, information that provides clues for the understanding of the efflux pump. These dynamically correlated hotspots could be of interest to find out targets of inhibitors. Moreover, steering only two of the three monomers led to results close to those when steering all three subdomains. This is certainly very interesting information when trying to understand the energy flow in the system. One could even speculate that the use of the proton gradient in at most two subdomains is enough for the full functional rotation of the whole complex. This would be consistent with current discussions in the scientific community that only two protons are needed per substrate extrusion or per full cycle. This hypothesis, however, still needs clear experimental proof.

## Figures and Tables

**Table 1:** Description of the TMD simulation setups used in this study, defined by selections of protein segments which are steered using the TMD approach. Some selections are a sum or difference of two other selections and are described accordingly. Note that each selection only refers to the *C*_α_ atoms of the individual amino acids.

Index	TMD selection	Time [ns]
*freeDyn *	None	200

*tmDom *	Transmembrane domain	50
*freeMon *	Neighboring monomers	50
*freePP *	*tmDom* + *freeMon *	50

*fullTMD *	Entire protein	50
